# Loneliness of the Blind and the Visually Impaired

**DOI:** 10.3389/fpsyg.2021.641711

**Published:** 2021-06-25

**Authors:** Ami Rokach, David Berman, Alison Rose

**Affiliations:** Department of Psychology, York University, Toronto, ON, Canada

**Keywords:** loneliness, blindness, visually impaired, alienation, social isolation, visually challenged

## Abstract

Loneliness has been termed a social epidemic, especially when experienced by people with disabilities. In order to better understand how loneliness is experienced in vulnerable populations, the present study compared the qualitative dimensions of loneliness of the blind and visually impaired with the general population not on the frequency or intensity of their loneliness, but on its *qualitative* aspects. One hundred and eighty-seven participants responded to a questionnaire which measured the qualitative aspects of loneliness on five subscales: Emotional distress, social inadequacy, Growth and discovery, social isolation, and emotional alienation. Results indicated that as expected, the two populations differed significantly in their scores on four of the five subscales (except emotional alienation), but in the opposite direction of what was expected. That may indicate that the visually impaired person’s ability to transcend their blindness, and connect with those around them, and the larger society, in different—and not necessarily less meaningful-manner than the seeing general population. As expected, the visually impaired scored significantly higher than the general population on the Growth and development subscale.

## Introduction

The present study, which adopted the biopsychosocial approach, sought to examine the quality of loneliness as affected by sight impairment or complete blindness. While there have been various research reports on the blind and their experiences, and those attesting to their loneliness which has been enhanced by their visual impairment (Osaba et al., 2019), no study to date has explored the qualitative dimensions of the loneliness that the visually impaired are experiencing.

There were many attempts, in the loneliness literature, to define the experience. Moustakas (1961) described loneliness based, almost solely, on his own and his clients’ experiences, while Peplau and Perlman (1982) theorized that loneliness exists in the difference between the amount of social contact that we experience and the amount we wish for. Peplau and Perlman (1982) tested their theoretical formulation utilizing the UCLA loneliness scale that aimed to measure the degree or depth of one’s loneliness. Cacioppo et al. (2000, 2002, 2003) and Hawkley et al. (2003) utilizing questionnaires and direct observations or measurement of physical variables, subscribed to Peplau and Perlman’s conceptualization of loneliness and furthered the field by researching how it is related to physical and mental health, to depression, to various social variables, and how it may even be “contracted” from one person in a group, to others. Salient threads in those definitions focus on deficiency of intimacy and meaning within a relationship, while others emphasize shortcomings in people’s sense of belonging and limited or lack of social connections (Stein and Tuval-Mashiach, 2015). In the beginning of the twenty-first century, people are apparently far more isolated than they were in the past. Social ties were an integral part of daily life in the past, but even prior to COVID having descended upon us, a growing number of people seem to have no (or very limited) close or intimate relationships and, thus, our society is increasingly fragmented (McPherson et al., 2006; Sha’ked and Rokach(eds), 2015). The Western culture, with its drive for individuality, magnifies the alienation and separateness that humans experience, while our basic need is to belong, to be needed and loved. Such a realization, is a salient cause of the awareness of our limitations and finality, resulting in loneliness (Rokach, 2019). Various attempts have been made to describe and define loneliness (i.e., Hawkley et al., 2003; Cacioppo et al., 2015; Petitte et al., 2015).

We examined how loneliness is described in the literature, and how it is commonly described by many theoreticians and researchers. In general, loneliness is defined as an experience in which we are alone, perceiving oneself as unloved, unimportant, and uncared for, and that leads to what we term loneliness (Martens et al., 2005; Rokach, 2019). There are some themes that come through the various theoretical orientations, and which are characteristic of loneliness experiences:

a Loneliness is an experience of separation, of one from one’s world.
b Loneliness may begin in childhood and remain throughout one’s life.
c It is associated with invalidation of one’s meaning and place in the world.
d Due to the pain and agony that it causes, it is difficult to tolerate.
e Loneliness appears to motivate humans to seek meaning and search for connection.
f It may have an evolutionary basis, in that loneliness motivates one to seek belonging which is protective and supportive; and
g Loneliness has the potential to enhance growth and new possibilities.

Based on the previous theoretical approaches, as well on his own research of the past four decades, Rokach (2019) pointed out three distinguishing characteristics of all loneliness experiences:

1 Loneliness is a universal phenomenon which is fundamental to being human.
2 Loneliness is a subjective experience that is subjected to personal and situational variables which affect it.
3 Loneliness, a multifaceted experience, is always very painful, highly distressing and individualistic.

Loneliness is a multifaceted experience which can be understood through the lens of the biopsychosocial model which is an interdisciplinary approach that looks at the interconnection between biology, psychology, and socio-environmental factors in affecting health, illness, psychological experiences and disturbances. Human development and experiences, according to this model, is multidimensional and is affected by the interaction of one’s biology, psychological processes, social interaction, culture and spirituality (Engel, 1981; see also Karl and Holland, 2015). The present investigation has adopted this approach to human experiences. To summarize it, we subscribe to the experiential approach put forth by Moustakas (1961) that loneliness is a natural experience in human lives, just as sadness, excitement, or hunger is as well. Our contribution to the literature (Rokach and Sha’ked, 2013) is that loneliness is like a recessive gene, which will be expressed and experienced as a result of one’s socio-environmental factors, health, psychological wellbeing or lack thereof, are culture and spirituality.

Visual impairment refers, generally, to poor vision. The term “*sight loss”* refers to people who have developed a visual impairment, when they have previously not had one. “*Blind”* is used for those who have no seeing capability at all. Visual acuity is the level of detail that can be seen by an individual. It may also be assessed by the extent to which an individual is able to perform daily activities for which sight is necessary, such as driving, reading and climbing up and down stairs (Hodge and Eccles, 2013).

Many people fear sight loss more than loss of any other sense (Baker and Winyard, 1998; De Leo et al., 1999). Those diagnosed with eye problems may experience anxiety, worry and concerns about the future, and even depression and increased emotional distress (Horowitz and Reinhardt, 2000; Scott et al., 2001; Norowzian, 2006). Quality of life can, significantly, be negatively affected by sight loss (Hassell et al., 2006) and can result in shock and grief (Evans, 1983; Baus, 1999). It was found that those who experience sight difficulties are more likely to feel lonely (Barron et al., 1992) since their social support is negatively affected to a greater degree than the general population (Bruce et al., 2007). Thurston et al. (2010) observed that isolation and the importance of social support are a frequent theme in the scientific literature about the visually impaired and those with sight difficulties (Cimarolli and Boerner, 2005; Percival and Hanson, 2007). Stephens (2007) as well as Horowitz and Reinhardt (2000) reviewed the pertinent literature and found that empirical research confirmed that visually impaired people experience difficulty in emotional functioning. Depression and reduced social activity as well as social support amongst the visually impaired were also reported, as well as a decrease in general wellbeing. For instance, in a study by Williams et al. (1998) on a sample of 86 people aged, on average, 79 years, and who had macular degeneration and vision of less than 10/30, mood was found to be lower in the visually impaired compared to elders who were sighted. Interestingly, even when compared to people with serious illnesses, the visually impaired scored lower on wellbeing scales. However, the difference in psychological distress did not reach significance when a sample of people with diabetic retinopathy was compared to those with no visual impairment (Upton et al., 1998).

A number of studies, exploring social connectedness of the visually impaired, reported that the progress of visual impairment was positively correlated with decreased levels of social functioning. In a large sample of 1,191 people with visual acuity less than 20/100, there was a positive correlation between visual impairment and lower social relationships (Carabellese et al., 1993). Similar findings were reported in Evans (1983) study on 84 registered blind participants who reported that their social activity decreased as the duration of blindness increased. In non-westernized settings, similar findings were found. Research on a sample of 117 Nepalese men, with visual acuity of less than 20/200, found that those people who had visual impairment were less likely to function as the head of their household, which is a highly valued role in that culture (Beall and Goldstein, 1986). Barron et al. (1992) found that impairment was positively correlated with loneliness in people with visual acuity less than 20/70.

In their qualitative study, Thurston et al. (2010) explored the process of becoming blind. Their sample included participants which were composed of 11 women and 7 men, average age of 65, all of whom had a recognizable condition which brought about loss of sight. Ten were registered blind, five were registered partially sighted and three were unregistered to date. None were blind from birth. Grounded theory was utilized to analyze the semi-structured interviews (Strauss and Corbin, 1990). Their analysis yielded a major category which they termed “making the transition to blindness.” Seven themes emerged from the open coding under this category: (1) with a diagnosis of a serious eye condition. (2) Addressing eyesight deterioration. (3) Experiencing loss. (4) Changes in perceptions of self. (5) Experiencing others. (6) Undergoing rehabilitation. (7) Being able to recognize things that are therapeutic.

Visual impairment (VI) represents a significant and many times irreversible loss in visual acuity or visual field. Visual impairment could either be congenital or acquired vision loss, and its severity could be moderate, severe, or total blindness (Colenbrander, 2010; International Statistical Classification of Diseases, and Related Health Problems 10th Revision, 2016). Since vision is a key sensory modality when it comes to interpersonal interactions, people with VI are particularly prone to loneliness. People who have severe VI or are totally blind have fewer opportunities to acquire appropriate social skills and may, thus, be prone to adverse interpersonal events which may result in loneliness (Jindal-Snape, 2004; Brunes et al., 2018a, b, 2019). Loneliness is a strong predictor of health and quality of life (Holt-Lunstad et al., 2015; Petitte et al., 2015; Beutel et al., 2017). Loneliness results in lower life satisfaction, compared to non-lonely people (VanderWeele et al., 2012). Brunes et al. (2019) conducted a cross-sectional study on a sample of adults with VI. It was found that almost 50% of adults with VI experienced moderate or severe loneliness, with higher rates across age groups, significantly more than the general population. Additionally, they found that the risk of loneliness was higher for people aged 36–50 years, who were also exposed to bullying or physical or sexual abuse, or suffered from other impairments. Lastly, as was previously established, their results indicated that intense loneliness was associated with lower life satisfaction.

Various studies have found that the disabled exhibit higher levels of loneliness than those without disabilities (McVilly et al., 2006; Coplan et al., 2007; White and Roberson-Nay, 2009). Research has found that loneliness experienced by students with disabilities is associated with deficient social skills and difficulties which may have been, consequently, experienced in peer relationships, especially during adolescence (Margalit and Ben-Dov, 1995; Yu et al., 2005). Hadidi and Al Khateeb (2013) investigated the association of loneliness and blind students in Jordan. Their sample was composed of 90 students with blindness (51 females) and 79 sighted students (44 females). Students’ age ranged from 15 to 22 years. It was found that, compared with sighted students, students with blindness experienced more loneliness. Several previous studies, carried out in other cultures, showed similar results and found higher levels of loneliness among blind students (for example, McGaha and Farran, 2001; George and Duquette, 2006). Possible causes for loneliness in students with blindness are perceived insufficient social support, duration of visual impairment, the manner in which the person adapted to vision loss, and depression (Barron et al., 1992; Verstraten et al., 2005). These results demonstrated that there were no significant gender differences in the intensity of the loneliness which study participants experienced (see Huurre, 2000; Kef, 2002). Additionally, the results found a significant correlation between age and the score of loneliness (see also Newman and Sachs, 2020).

### The Present Study

The literature points to a significant occurrence of loneliness in people who are sight challenged. The present study did not aim to measure the prevalence or intensity of loneliness but the *qualitative dimensions* of it. Attempting to ease their isolation and sense of alienation may be helped and improved if we can gain an understanding of what they actually experience, as a corollary of their loneliness. For the present study and based on previous research which highlighted the more intense loneliness of the blind and visually impaired, it was hypothesized that the blind sample would report significantly higher scores on all qualitative subscales of loneliness as compared to the rest of the general population, To clarify, it was not hypothesized that the blind group would experience a higher intensity of loneliness than the general population, but that they would score higher on each of the scales measuring the qualitative aspects of loneliness.

## Materials and Methods

### Participants and Procedure

A total of 187 participants from the general population volunteered to, anonymously, answer the loneliness questionnaire. In this study, which was carried out in urban centers in Israel and which received approval from the university’s IRB, participants were asked to mark those items which described their past loneliness experiences. As can be seen in Table 1, the sample was predominantly composed of females, which were more available and agreeable to participate. Those of the general population took approximately 10 min to answer the questionnaire. They were recruited, via snowball technique, in community centers with the permission and support of gatekeepers who encouraged attendees to participate. Participants were given the questionnaire in person or interviewed on the phone if they so preferred, where they were asked to respond to the questionnaire. The visually impaired or the blind (hence referred to as the “blind” group) were contacted either in centers for the blind again with the assistance of gatekeepers, or via the phone, and again utilizing the snowball technique. These participants were read the questionnaires and those items that represented their experiences, thoughts, or feelings regarding loneliness were marked. All participants were provided a verbal consent form, which they all agreed with and endorsed, and were promised anonymity. Data were gathered for approximately 4 months, during the end of 2018.

**TABLE 1:** Demographics.

Population	*N*	Marital status			Education		Age	
		Single	Married	Divorced/Separated widowed	*M*	*SD*	*M*	*SD*
Blind	65	32 (49%)	21 (32%)	12 (18%)	12.2 (12–16)	0.74	44.5 (18–85)	23.5
Men	22 (34%)	13	8	1	12.2	0.853	37.60	19.5
Women	43 (66%)	19	13	11	12.2	0.0679	48.00	24.8
	*X*^2^_(__1,__2)_ = 4.315				*F*_(__1,__58)_ = 0.0143		*F*_(__1,__63)_ = 2.92	
General Pop	122	64 (52%)	52 (43%)	6 (5%)	13.3 (11–19)	1.48	28.2 (19–57)	8.72
	12 (10%)	7	5	0	13.5	1.45	31.4	8.78
	110 (90%)	57	47	6		1.48	27.9	8.69
	*X*2_(__1,__2)_ = 0.745				*F*_(__1,__120)_ = 0.216		*F*_(__1,__120)_ = 1.77	
Total	187	96 (51%)	73 (39%)	18 (10%)	12.9	1.39	33.9	17.3

### Measure

The Loneliness Questionnaire includes 30 yes/no items, where participants were asked to endorse those statements which describe their experience of loneliness. All items for the questionnaire are based on Rokach’s previous research on loneliness (Rokach, 1988a, b; see also Rokach and Brock, 1997; Chin et al., 2013). The questionnaire has been in use for over three decades. Rokach (1988a) study was based on responses of five hundred and twenty-six (526) subjects who described their experiences of loneliness. The items for the questionnaire utilized in the present study were chosen from those descriptions and were modified to provide clarity and gender neutrality. The questionnaire is unique in that, unlike all other loneliness measures (i.e., UCLA’s loneliness questionnaire) it does *not* measure the intensity or frequency of experiencing loneliness, but rather the qualitative dimensions of that experience. As such, and since it was based on qualitative and quantitative analyses of a very large sample that described their loneliness experience, it cannot be compared to any other measure of loneliness for, say, validation purposes. The questionnaire was developed based on participants’ responses about their experience of loneliness, in a cognitive, emotional, and behavioral sense.

When the questionnaire was constructed, in the mid-1980s, principal components factor analysis with varimax rotation was applied to the data with 0.40 being the minimum loading for an item. Using an SPSS program, we extracted the principal components by factor analysis, and the factor matrix was then subjected to varimax rotation. Five factors emerged, and each accounted for at least 3% of the variance to support statistical meaningfulness. Accordingly, repetitions of the varimax rotations were limited to five factors each to permit the results to be restricted to the most roust factors.

That analysis yielded five factors. Emotional distress (which accounted for 19% of the variance) was the most salient factor. It included items that captured the intense pain, the hopelessness, and the feelings of emptiness which are so prevalent in loneliness. The second factor, Social inadequacy and alienation (7% of the variance) focused on the self-generated social detachment which many reported as part of their loneliness experience. Growth and discovery (4% of the variance) was the third factor. It highlighted the growth-enhancing, and enriching aspects of loneliness and the increased inner strength and self-reliance that the person may experience. Interpersonal isolation (3% the variance) was the fourth factor. This factor described the feelings of alienation, and rejection, which were related to lack of close relationships and/or absence of a primary romantic relationship. The fifth factor, Self-alienation (3% of the variance) captured the detachment from one’s self which could be experienced together with numbness, immobilization and denial. Each subscale in the questionnaire was based on a factor and participants’ scores are the sum of items which they endorsed in each subscale. Thirty items composed the questionnaire, and each of the five scales had six items (see Rokach, 2000). Participants were asked to reflect on their experience of loneliness and endorse the items that describe them.

This scale captures more diverse facets of the loneliness experience than other loneliness measures that merely assess social and emotional loneliness. It has demonstrated adequate psychometric properties, with high reliability (e.g., Orzeck and Rokach, 2004). Since the loneliness questionnaire was developed using a phenomenological approach whereby more than 660 participants were surveyed for the efforts of capturing a valid description of the experience of loneliness that was also as comprehensive as possible. Additionally, there is no other measure, to our knowledge, which examined the qualitative (rather than quantitative) aspects of loneliness, and it is thus impractical to validate it in comparison to any other scale. In the current sample, the following Kuder-Richardson internal consistency reliability values were found: Emotional distress = 0.75; Social inadequacy and alienation = 0.71; Growth and discovery = 0.88; Interpersonal isolation = 0.70; Self-alienation = 0.59. The reliability for the entire questionnaire was 0.87 in the current sample. The questionnaire was translated into Hebrew by two bilingual Canadian students and was then verified and edited by an Israeli academician.

### Statistical Analysis

Statistical analyses were conducted with SPSS version 27^1^. Descriptive statistics were examined for the study variables (see Table 1). First, chi square analyses assessed whether there were any differences within the groups (general population or blind individuals) with regards to marital status. Additionally, tests using analysis of variance (ANOVA) evaluated whether there were significant differences within the groups with regards to both education and age. Next, multivariate analysis of variance (MANOVA) was used to test whether groups overall significantly differed in their loneliness experience, either based on population (blind individuals and general population) or gender (men and women). Depending on whether an overall MANOVA was significant, subsequent ANOVAs were run to test whether scores on the five factors of the loneliness questionnaire differed significantly between population or gender group.

## Results

The sample was comprised of 34 men and 153 women aged between 18 and 85 years, with a mean age of 33.9 years (*SD* = 17.3). Of the total sample, 65 individuals identified as blind or severely visually impaired (22 men), whereas 122 individuals identified as seeing folks (12 men, and 10 women). Education (i.e., last grade completed) was, on average 12.9 years (range 11–19). Fifty one percent of the participants were single, 39% married, and 10% had a relationship but were no longer in it due to separation, divorce or death of a spouse. Table 1 provides a more detailed breakdown of gender, age, education and marital status within each of the two population groups, namely the blind and the general population. As depicted by the results in Table 1, there were no significant differences within the groups with respect to marital status, education, or age, and thus none of these variables needed to be incorporated as covariance within further analyses.

The mean subscale scores for participants in each group and for each factor are shown in Table 2. Table 2 illustrates that there were significant differences between the blind and general population groups. The mean subscale scores for participants in each group and for each factor are shown in Table 2. An overall MANOVA [*F*(5, 181) = 8.79; *p* < 0.001] indicated that the two populations differed significantly in their loneliness experience. ANOVAs for each subscale indicated that on all subscales, except for the self-alienation, the groups differed significantly. On all subscales, but Growth and discovery, the mean subscale scores of the general population were significantly higher than those of the blind.

**TABLE 2:** Mean subscale scores and f ratios for the populations under study.

Population	*N*	Emotional distress		Social inadequacy		Growth and discovery		Social isolation		Self-alienation	
		*M*	*SD*	*M*	*SD*	*M*	*SD*	*M*	*SD*	*M*	*SD*
Blind individuals	65	2.29	1.96	2.10	1.59	3.69	2.37	1.83	1.82	1.43	1.38
Men	22	2.54	1.92	1.95	1.58	4.36	2.21	2.13	1.90	1.86	1.39
Women	43	2.16	1.99	2.18	1.60	3.34	2.40	1.67	1.78	1.20	1.33
MANOVA	*F*_(__5,__59)_ = 2.39	*F*_(__1,__63)_ = 0.54		*F*_(__1,__63)_ = 0.30		*F*_(__1,__63)_ = 2.72		*F*_(__1,__63)_ = 0.93		*F*_(__1,__63)_ = 3.39	
General Pop	122	3.35	1.84	2.82	1.93	2.43	2.26	2.91	1.75	1.79	1.60
Men	12	2.75	1.95	3.00	1.80	1.33	2.01	2.75	1.54	1.41	1.50
Women	110	3.41	1.82	2.80	1.95	2.55	2.26	2.93	1.77	1.83	1.61
MANOVA	*F*_(__5,__116)_ = 1.14	*F*_(__1,__120)_ = 1.43		*F*_(__1,__120)_ = 0.10		*F*_(__1,__120)_ = 3.19		*F*_(__1,__120)_ = 0.12		*F*_(__1,__120)_ = 0.74	
Men	34	2.61	1.90	2.32	1.71	3.29	2.57	2.35	1.79	1.70	1.42
Blind individuals	22	2.54	1.92	1.95	1.58	4.36	2.21	2.13	1.90	1.86	1.39
General Pop	12	2.75	1.95	3.00	1.80	1.33	2.10	2.75	1.54	1. 41	1.50
MANOVA	*F*_(__5,__28)_ = 6.01**	*F*_(__1,__32)_ = 0.08		*F*_(__1,__32)_ = 3.05***		*F*_(__1,__32)_ = 15.44		*F*_(__1,__32)_ = 0.91		*F*_(__1,__32)_ = 0.75	
Women	153	3.06	1.95	2.63	1.88	2.77	2.32	2.58	1.86	1.66	1.56
Blind individuals	43	2.16	1.99	2.18	1.60	3.34	2.40	1.67	1.78	1.20	1.33
General Pop	110	3.41	1.82	2.80	1.95	2.55	2.26	2.93	1.77	1.83	1.61
MANOVA	*F*_(__5,__147)_ = 5.99***	*F*_(1_, _151)_ = 13.86***		*F*_(__1,__151)_ = 3.44		*F*_(__1,__151)_ = 3.65		*F*_(__1,__151)_ = 15.55***		*F*_(__1,__151)_ = 5.12	
Totals	187	2.98	1.94	2.57	1.85	2.87	2.37	2.54	1.84	1.66	1.53
Blind individuals	65	2.29	1.96	2.10	1.59	3.69	2.37	1.83	1.82	1.43	1.38
General Pop	122	3.35	1.84	2.82	1.93	2.43	2.26	2.91	1.75	1.79	1.60
Total MANOVA	*F*_(__5,__181)_ = 8.79***	*F*_(__1,__185)_ = 13.41***		*F*_(__1,__185)_ = 6.60*		*F*_(__1,__185)_ = 12.61***		*F*_(__1,__185)_ = 15.87***		*F*_(__1,__185)_ = 2.41	

*^∗^p < 0.05 ^∗∗^p < 0.01 ^∗∗∗^p < 0.001.*

Further, while there were no significant differences within the two populations between men and women, when compared by gender, men from the general population differed significantly from those of the blind group [F(5, 28) = 6.01; *p* < 0.01]. That difference was related to the significant difference between the two groups on the Social inadequacy subscale [*F*(1, 32) = 3.05; *p* < 0.001]. Comparing the women in the two population groups, there was once again an overall significant difference [*F*(5, 147) = 5.99; *p* < 0.001]. That difference was related to significant differences in three of the five subscales, namely Emotional distress, Social isolation, and self-alienation where in all three the general population women scored higher than their blind counterparts.

## Discussion

The present study examined the qualitative aspects of people with blindness or significant sight challenges, by comparing them to that of a comparable sample of the general population. It was emphasized, by previous research, that serious illness or disability such as blindness, is related to loneliness and perceptions of social isolation (Martens et al., 2005; Rokach et al., 2006; Rokach, 2007). Increasing our understanding of the loneliness experience of those who are visually challenged, may enhance society’s ability in assisting them to overcome it, or at least ease their pain and sense of isolation.

The current authors adopted the biopsychosocial approach to health, illness, and disability, thus recognizing the social, psychological, physiological and spiritual aspects which interact and affect health or lack thereof (Brannon and Feist, 2004). The present study focused on people with sight disabilities, which by their nature may cause problems related to dexterity or movement, vision or hearing and negatively affect their communication with others (Robinson et al., 1995). While many may experience various minor losses such as a dent in a new car or being stuck in a traffic jam, such temporary losses occur throughout life and inflict anxiety and disappointments. Yet, they are reversible and eventually not considered to be significant. Serious losses, such as sight difficulties or blindness are the most painful losses to bear. Such permanent losses may, according to Robinson et al. (1995), violate one’s identity and negatively affect a person’s dreams and hopes. Kennedy (1999) referred to the architectural and social inaccessibility of our society, which is geared for able-bodied people, as the “disabling environment” which may magnify the difficulties and sense of not belonging for the visually challenged person (p. 138).

Our results indicated that in each of the two population groups, the quality of men’s loneliness does not seem to differ from that of women’s. Hodge and Eccles (2013) explored gender differences regarding the experience of loneliness and also found that the genders did not differ in the rates of experienced loneliness in the visually impaired. When addressing the *quality* of the loneliness experiences of the two genders, results of the present study may indicate that the loneliness questionnaire which we utilized addressed issues, concepts and experiences which are experienced similarly by both genders, meaning that qualitatively loneliness of either gender is experienced similarly. It is therefore suggested that the loneliness questionnaire is comprehensive in that it encapsulates all if not most of qualitative aspects of loneliness, including gender differences.

Generally, we found that the visually impaired experience loneliness differently than the general population. Brunes et al. (2019) conducted a large-scale study on 736 visually impaired and blind participants in Norway and found that their loneliness was greater, more intense, than that of the general population. He noted that since those with visual impairment are prone to risk of disability, poor health, low financial income, and difficulties in interpersonal events, all may bring about or deepen one’s loneliness. Interestingly, our results indicated that the visually impaired had significantly *lower* scores on all subscales but the Growth and development one. We would intuitively expect that those who may feel so poignantly different from the rest of the population, who live using other senses instead of sight which is a major sense used by non-visually impaired individuals (see Osaba et al., 2019), may be experiencing social inadequacy in a world which is not, fundamentally, geared to accommodate them, and thus may feel alienated, to a larger extent than the regular population (see Nenov-Matt et al., 2020 for a discussion of the loneliness-social isolation interaction). However, the present findings showed a significantly different picture, where the general population had significantly higher subscale scores than the visually impaired. That may indicate that the visually impaired person’s ability to transcend their blindness, and connect with those around them, and the larger society, in different—and not necessarily less meaningful-manner than the seeing general population. Additionally, it is suggested that, as the Growth and development subscale indicates, the blind are more attuned to their inner world, and with less distractions of the outside environment, may be more open to enhance their self-appreciation, and increase their self-confidence and personal strength.

While visually impaired individuals may experience *higher or more intense* loneliness as previous research indicated, their loneliness may not differ, qualitatively, from that of the general population. Osaba et al. (2019) maintained that blindness, and visual impairment precipitate depression, insecurity, anxiety, changes in social functioning, and loss of independence. Pinquart and Pfeiffer (2014) echoed that finding and mentioning that a meta-analysis found increased psychological distress in individuals with visual impairment as compared to sighted peers. It is, thus, suggested that while the visually impaired may be more prone than the general population to experience loneliness, they attributed their emotions, cognitions and behaviors, to their visual disability rather than to loneliness. In other words, as Brunes et al. (2019) and Osaba et al. (2019) have stated, navigating and completing daily activities as a blind or visually impaired person is much more challenging than if one possessed good sight. Consequently, it is suggested that the visually impaired, when asked to indicate the quality of their experience of loneliness, may attribute them to their impairment, rather than to their loneliness, which was attested to by their lower subscale scores. Horowitz and Reinhardt (2000) described the difficulty in emotional functioning amongst visually impaired people. They pointed to depression, losses of social activity, reductions in social support amongst visually impaired people as well as decreases in general wellbeing. Living in such turmoil, it may stand to reason that the individual will see the blindness, and not one’s loneliness, as related to the dissatisfaction, aloneness, and emotional turmoil, rather than one’s loneliness.

When examining the rest of the present results as displayed in Table 2, one can see that, consistently, males and females from the general population had higher subscale scores than their counterparts from the blind population. Interestingly, when visually impaired males were compared to those of the general population, the only significant difference in their subscale scores was on the Social inadequacy subscale. We propose that males in the general population are facing social pressures and feel a need to appear socially adequate in order to be able to earn a living, push their way up the social ladder, and create and maintain a social support network. The pressure may be less for visually impaired individuals who are significantly disabled and may, thus, garner empathy and compassion from those around them, without needing to appear so able and socially adequate, resulting in a lower social inadequacy subscale score (see also Taylor, 2012). That, naturally, is a tentative explanation since the number of males who participated in the present study is smaller than we would hope for.

Comparing visually impaired women to their counterparts in the general population, we found that visually impaired women had significantly lower subscale scores on the Emotional distress and Social isolation subscales. Research that we previously mentioned (i.e., Carabellese et al., 1993; McVilly et al., 2006; White and Roberson-Nay, 2009), confirmed that blindness increases one’s emotional suffering. However, it is possible that visually impaired women, while they are impacted by loneliness due to their impairment, assign their emotional turmoil to their visual challenges rather than to their loneliness. When responding to items in our questionnaire such as “when I am lonely, I experience feelings of intense hurt” or “It feels like my heart is breaking” the visually impaired woman may have experienced such feelings and pain, but in response to her blindness and *not* loneliness.

The remaining results did show significant differences between the population groups or genders. However, one non-significant result of interest regarding the Social-alienation subscale, which evaluates detachment from the self potentially resulting in emotional numbness, immobilization, and denial, revealed itself. As far as we can ascertain, it is unexplainable that both populations did not significantly differ on this particular subscale, although the visually impaired may, due to their condition, be familiar with numbness and immobilization which may accompany difficulties, frustrations, and struggles that are experienced (see also Thurston et al., 2010; Hodge and Eccles, 2013).

### Study Limitations

The present study utilized a snowball technique for recruiting participants and was based on a convenience sample, where potential participants were approached in community centers, parks, or shopping malls and asked to anonymously participate. Thus, while we attempted to sample as wide a sample of the general population, it was not done in a systematic manner and generalizability of the results may be limited in scope. Moreover, the sample was predominantly composed of females, which were more available and agreeable to participate, thus highlight a need for further research which would include a larger number of males. Our study did not differentiate those with visual impairment, from the severely visually impaired, or from those who were completely blind, nor did we gather information on the length of the visual impairment which the participants experienced. That variable, naturally, may have affected the experience of loneliness. Given that the key measure in the study was developed in the mid-1980s, we also acknowledge that there could be merit in further evaluating the measure and assessing it with more updated statistical techniques, such as revising the scale to a continuous response format and again undergoing the factor analytic procedure. In this way, the incremental validity of the Loneliness Questionnaire beyond other existing measures (i.e., the UCLA Loneliness Scale) could be a key focus for future evaluation, although this is beyond the scope of the present research.

Additionally, the present study did not explore the professions of the blind. It is suggested that this variable could also affect the way that the visually impaired view their social relations and integration within the fabric of society. There are organizations, and clubs that offer social opportunities as well as aids to compensate for sight difficulties. These resources may enhance their social contacts and social support network, and could affect the intensity, quality, and length of their loneliness experience. In the present study we did not investigate whether our participants belonged to such clubs or organizations. Additionally, it is suggested that the length of time that a person suffers from visual impairment, and whether it was an abrupt blindness or a gradual one, may affect the person’s adaptability and feelings of alienation. Regarding the loneliness questionnaire, like any other measure, there is merit in further evaluating the measure and assessing it with other statistical techniques (including changing it to a continuous response format) and that of course, as in other studies, the results reflect the strengths and limitations of the main measure used. In this instance, the incremental validity of the Loneliness Questionnaire beyond other loneliness measures (e.g., the UCLA Loneliness Scale) is a key focus for future evaluation.

### Implications and Impact of the Current Research

To conclude, the present study explored the inner world of the blind and the visually impaired by aiming to understand the quality of the loneliness which they may experience. Results indicated that while there were no significant differences between genders in the blind population, visually challenged women significantly differed in their experience of loneliness from women in the general population. In general, the two population groups under study differed significantly on four of the five subscales, mostly in the opposite direction of what we expected, except where the blind had significantly higher subscale scores on the Growth and discovery subscale. This could be interpreted as a testament to their resilience and their ability to compensate and overcome their disability which is widely considered a serious impediment to a full life. The present results will, hopefully, spur further research which could aid in enriching the inner and social worlds of the blind.

## Data Availability Statement

The raw data supporting the conclusions of this article will be made available by the authors, without undue reservation.

## Ethics Statement

The studies involving human participants were reviewed and approved by the Institutional Review Board, Center for Academic Studies, Israel. Written informed consent for participation was not required for this study in accordance with the national legislation and the institutional requirements.

## Author Contributions

AmR conducted the study and wrote most of the manuscript. DB did statistical analyses together with AlR. All authors contributed to the article and approved the submitted version.

## Conflict of Interest

The authors declare that the research was conducted in the absence of any commercial or financial relationships that could be construed as a potential conflict of interest.

## Footnotes

1 https://www.ibm.com/ca-en/products/spss-statistics
